# Knowledge gaps in psychedelic medicalisation: Preclinical and neuroimaging mechanisms

**DOI:** 10.1016/j.nsa.2023.103929

**Published:** 2023-12-15

**Authors:** Drummond E-Wen McCulloch, Juan Pablo Lopez, Christina Dalla, Eero Castrén, David Erritzoe, Vibe G. Frokjaer, Johan Lundberg, Katrin H. Preller, Patrick MacDonald Fisher, Gitte Moos Knudsen

**Affiliations:** a Neurobiology Research Unit, Rigshospitalet, and University of Copenhagen, Copenhagen, Denmark; b Department of Neuroscience, Karolinska Institute, Solna, 171 77, Sweden; c Department of Pharmacology, Medical School, National and Kapodistrian University of Athens, Athens, Greece; d Neuroscience Center, HiLIFE, University of Helsinki, Helsinki, Finland; e Centre for Psychedelic Research, Department of Brain Sciences, Imperial College London, London, UK; f Institute of Clinical Medicine, University of Copenhagen, Copenhagen, Denmark; g Centre for Psychiatry Research, Department of Clinical Neuroscience, Karolinska Institutet, & Stockholm Health Care Services, Region Stockholm, Karolinska University Hospital, SE-171 76, Stockholm, Sweden; h Pharmaco-Neuroimaging and Cognitive-Emotional Processing, Department of Psychiatry, Psychotherapy and Psychosomatics, Psychiatric University Hospital, University of Zurich, Zurich, Switzerland; i Department of Drug Design and Pharmacology, University of Copenhagen, Copenhagen, Denmark

## Abstract

Classical psychedelic drugs, e.g., psilocybin and LSD, stimulate the serotonin 2A receptor (5-HT2AR) and have recently been intensely investigated for their clinical effects in various brain disorders. At the ECNP “New Frontiers meeting” in March 2023, scientific experts in psychedelics met to identify key knowledge gaps in the mechanism of action of psychedelics as investigated using preclinical models and clinical neuroimaging. Key themes included the development of appropriate behavioural models for measuring acute and persisting effects, dose optimisation, molecular mechanisms of action, sex differences, and the acute and persisting effects of psychedelics on neurotransmitter release and functional brain activity.

## 1 Introduction

Classical psychedelic drugs are biased serotonin 2A receptor (5-HT2AR) agonists, which produce dose-dependent LSD-like subjective effects in humans. Since the turn of the century, several clinical trials administering psychedelics have been performed, showing early promise for the treatment of psychiatric conditions including depression (Carhart-Harris et al., 2021; Goodwin et al., 2022), cigarette and alcohol addiction (Bogenschutz et al., 2022, Johnson et al., 2017) as well as adjustment disorders (Anderson et al., 2020; Griffiths et al., 2016). With this in mind it was considered timely by the European College of Neuropsychopharmacology (ECNP) to host a “New Frontiers meeting” (referred hereafter as “the meeting”) in April of 2023 to discuss the path forwards for psychedelic drugs as medicines. Here, academics, clinicians, industry and regulators gathered to discuss the most pressing gaps in psychedelic medicalisation at this point.

The exact mechanisms of therapeutic action of psychedelic drugs have not yet been resolved, and there are several competing theories being tested. In part 1 of this perspective, we describe the knowledge gaps highlighted at this meeting and identify forward-looking mechanistic perspectives on the development and optimisation of psychedelic medicine. Identification of the mechanism of action of psychedelic therapy is essential for optimising the safety, efficacy and cost-effectiveness of current implementation of known psychedelic drugs as medicines, as well as in the development of novel related compounds. In this perspective, we focus on preclinical research and clinical neuroimaging as tools for uncovering the mechanism of therapeutic action of psychedelics, and in a subsequent perspective we focus on clinical and regulatory aspects.

## 2 Preclinical methods

Emerging new studies using preclinical models to investigate the molecular mechanisms and behavioural correlates of treatment response are promising, but there are several areas where pre-clinical psychedelic research should be further explored. More specifically, (1) use of preclinical models to optimise dose-selection for future clinical work (2) implementation of relevant preclinical models to assess treatment response (responders vs non-responders), (3) investigation of the molecular mechanisms and mode of action, and (4) role of sex differences. Standardisation of methods and pharmacological interventions will increase reproducibility and comparisons across different studies. Future preclinical studies and protocols should produce robust data and enhance overall quality of neuropsychopharmacological research. “PEERS”, an open-access Platform for the Exchange of Experimental Research Standards in Biomedicine could be used for this cause (Sil et al., 2021).

### *2.1* *In vitro* dose-optimisation

The meeting was well represented by drug companies that currently are either working with well-known classical psychedelics or are developing novel 5-HT2AR ligands. Ensuring target interaction and choosing the right dose for subsequent larger clinical trials is instrumental for a successful outcome of such studies. The choice of a dose that is too low will reduce the effect size and if too high, adverse effects may prevent the compound from making it into the clinic. The classical psychedelics represent a particular case because they produce clear subjective effects, measurable using scales such as the 5-dimensions of altered states of consciousness questionnaire, and physiological effects objectively verifiable in terms of increased mean arterial blood pressure, heart rate, body temperature and pupillary dilatation. Having access to both subjective and objective measures is possibly advantageous for selecting doses, yet there is only limited evidence for which qualitative and quantitative aspects of the psychedelic experience are required (if at all) for treatment efficacy. Of note, ongoing work on identifying 5-HT2AR compounds with biased agonism and with neuroplastic effects but without the psychedelic effects do not conform to such an approach. Below, we discuss some of the approaches commonly used to identify the right dose of a compound, particularly for novel compounds where less is known.

For novel potential psychedelic compounds under development, in vitro 5-HT2AR binding affinity (Ki) and half-maximal inhibitory concentration (EC50) can provide a first estimate of the dose needed in vivo to elicit psychedelic effects (Luethi and Liechti, 2018). 5-HT2AR binding generally correlates with the human psychoactive dose, and even better when taking pharmacokinetic properties into account. Additionally, subjective drug intensity has been shown to be closely linked to 5-HT2AR occupancy in humans (Madsen et al., 2019). However, affinity values determined in vitro in different laboratories cannot be directly compared. Therefore, in vitro profiling of a novel compound needs to include known classical psychedelics like psilocin, N,N-dimethyltryptamine (DMT), or LSD. For in vitro studies, also in its radiolabelled form, the 5-HT2AR antagonist MDL 100,907 (volinanserin) may be superior when high 5-HT2AR selectivity is desirable. Unwanted off-target binding (HERG, 5-HT2BR) can be assessed in vitro. The contributing role to the subjective psychedelic effects of targets other than the 5-HT2AR are considered limited, since the acute psychedelic experience largely can be blocked with ketanserin (Becker et al., 2022; Holze et al., 2021; Preller et al., 2017; Vollenweider et al., 1998). It is theoretically possible for non-5-HT2AR binding to modify the effects initiated by 5-HT2AR agonism, but there is no direct evidence for this, except a single small study indicating that pre-treatment with pindolol, a 5-HT1AR antagonist, perpetuated psychedelic effects (Strassman, 1995). If non-5-HT2AR targets mediate some aspects of the response, such as TrkB mediating neuroplasticity (Moliner et al., 2023), these will most likely not be captured by assessment of 5-HT2AR affinity.

### *2.2* *In vivo* dose-optimisation

The head-twitch response (HTR) in animals is generally assumed to reflect a psychedelic experience and to confirm that the drug is a 5-HT2AR agonist which may have psychedelic effects (Halberstadt and Geyer, 2013). Although it is not clear how well this behaviour translates to human psychedelic experiences, the construction of a dose-HTR curve may be useful for eventual human dose-selection. Direct translation to human dose-selection is not straightforward as rodent doses do not easily convert to humans. Although formulae are available (Nair and Jacob, 2016), further work contrasting HTR and human dose-response curves will further elucidate if such formulae are applicable to the conversation between HTR and clinical subjective effects. However, by including in parallel dose-ranges of psychedelics with known dose-response profiles in humans, such as LSD and psilocybin, doses may be determined that can be taken into phase-1 clinical trials. A similar approach can also be applied to drug-discrimination studies to evaluate the threshold dose at which a novel psychedelic may substitute for a known psychedelic (Baker, 2018).

It is unclear to which extent the head-twitch response correlates with serotonin-receptor occupancy, antidepressant effects and plasticity effects. Animal 5-HT2AR occupancy studies have so far not been performed much; (Tsartsalis et al., 2021) one study describes the relation between the head-twitch response induced by agonists and 5-HT2AR occupancy in rats (Kiilerich et al., 2023), and another shows that pigs also display a head-twitch response when given intravenous psilocybin at doses that correspond to about 67% 5-HT2AR occupancy (Donovan et al., 2021) an IC50 of about 7 μmol/L of psilocin (unpublished, Knudsen), which is close to the 2 μmol/L observed in humans (Madsen et al., 2019). The pig, with its larger and gyrated brain, may thus under certain circumstances be considered as an appealing alternative to occupancy studies in humans.

### 2.3 Behavioural models

In the context of neuropsychiatric disorders, most animal studies investigating the response to treatment are based on analyses using standard behavioural paradigms to assess anhedonia and despair-like behaviours, such as the forced swim, tail suspension, or glucose preference tests, among others (Planchez et al., 2019). While their simplicity is advantageous, the face and construct validity of these commonly used tests have significant limitations (Gururajan et al., 2019). These tests are ethologically questionable, limited to detailed observations of a single arbitrary measurement in a short period of time, ignore social and individual characteristics of rodents, and require significant intervention by the experimenter (Planchez et al., 2019). The social preference test is often used to assess social deficits in mouse models of autism (Rein et al., 2020), but has also been applied in studies of psychedelics. To improve on these issues and to tackle questions regarding response to psychedelic treatment, new behavioural methods should be developed and standardised to assess response to treatments. The head-twitch response in animals is commonly used as a proxy to infer a psychedelic experience and 5-HT2AR agonism (Wulff et al., 2023). However, this particular behaviour tells us very little about response to antidepressant treatment. New behavioural paradigms to assess response to treatment have been developed. For example, during the meeting, Dr. Emma Robinson described a new method to behaviourally characterise response to pharmacological treatments, including psilocybin, using a judgement bias task to test how rats respond to emotional and ambiguous stimuli under different affective states (Jones et al., 2018). Another proposed assay put forward by Dr. Gül Dölen investigates changes in the social reward learning critical period, which has been found to be re-opened following psychedelic drug exposure (Nardou et al., 2023). While these methods represent a good step forward, they are low throughput, requiring significant intervention and training by the experimenter. A potential improvement is the implementation of semi-naturalistic and automatic phenotyping systems to assess treatment response in animal models (Miranda et al., 2023). These emerging methodologies using advanced computer vision and machine-learning techniques offer novel, open-source methods for standardised pose estimation that enable continuous and automatic tracking of individual body parts over long periods, with minimal intervention by experimenters (Shemesh and Chen, 2023). For example, using a combination of supervised and semi-supervised classifiers to automatically quantify social interactions and hierarchies among groups of mice, Lopez et al., developed a classifier to assess response to ketamine treatment in mice (Lopez et al., 2022). Moreover, these methods should be performed in combination with animal models of stress (von Mücke-Heim et al., 2023; Hodes et al., 2023), substance abuse (Venniro et al., 2020), as well as autism disorders (Silverman et al., 2010) to further study the relevant effects of psychedelic compounds. These include unpredictable chronic stress models to induce depression-like symptoms, operant and non-operant models of drug-taking behaviours, as well as social preference tests to assess social deficits in mouse models of autism (Kokras and Dalla, 2014). New efforts to develop and standardise methods to evaluate treatment response in a multimodal way can help disentangling individual differences between responders and non-responders to treatment, which can lead to the identification of novel mechanisms and modes of action for psychedelic compounds.

### 2.4 Molecular mechanisms of action

A key outstanding knowledge gap concerns the molecular pathways through which psychedelics produce lasting reductions in clinical symptoms. During the meeting, Dr. David Olson presented exciting new data, using in vitro and in vivo models, to suggest a novel mechanism of action for psilocybin via activation of the intra-cellular 5-HT2A receptors to mediate the plasticity-promoting properties of psychedelics (Vargas et al., 2023). Additionally, psychedelic-induced plasticity via direct binding to the BDNF receptor TrkB as a positive allosteric modulator has been recently proposed as a mechanism through which they produce their therapeutic effects (Moliner et al., 2023). There is a widespread agreement that changes in neuronal plasticity are at least a component in the mechanism of psychedelic action, especially in the long-lasting effects (Kwan et al., 2022). Neurite outgrowth and spinogenesis has been widely studied both in vitro and in vivo to assess the effects of psychedelics on neuronal plasticity (Vargas et al., 2023; Cameron et al., 2023; Jefferson et al., 2023; Ly et al., 2018, 2021; Shao et al., 2021), but these methods cannot be used in humans. Plasticity of sensory or TMS-evoked responses (Normann et al., 2007; Voineskos et al., 2019) has been successfully demonstrated in humans using electroencephalography (EEG), but it is unclear whether these responses correspond to the state of plasticity that can be induced by antidepressants in rodents (Maya Vetencourt et al., 2008). A recent study demonstrated that psilocybin induces a long-term increase in synaptic vesicle density in the pig brain (Raval et al., 2021), using PET imaging of synaptic vesicle protein 2A (SV2A), a marker of synaptic vesicle density that has been linked to plasticity (Mak et al., 2021; Thomsen et al., 2021). These findings indicate that in vivo methods might be available soon to investigate states of plasticity and their modulation by psychedelics though there remain concerns about the specificity of neuroplastic effects to psychedelic drugs and to their causal relation to improvements in clinical symptoms. Future animal behavioural work as highlighted above may be complemented by the addition of in vivo molecular imaging using PET and autoradiography and radiotracers such as [11C]Cimbi-36 (Finnema et al., 2014) and [11C]PHNO (Tziortzi et al., 2011) to investigate persisting alterations in serotonergic and dopaminergic function respectively.

### 2.5 Sex differences

Although sex differences in treatment efficacy can be determined clinically, preclinical research can complement clinical work by allowing for closer examination of the impact of sex-relevant variables such oestrous cycle phase or reproductive periods (e.g. gestation, post-partum, senescence) as well as allowing for evaluation of outcomes not measurable in clinical populations, for example Cameron et al., have shown that repeated doses of DMT are associated with decreased dendritic spine density in the prefrontal cortex of female, but not male rats (Cameron et al., 2019). Findings from preclinical studies can also contribute to the better design of clinical trials, which will take into consideration the role of sex/gender and possibly result in gender-tailored treatments (Rechlin et al., 2022). Open-access videos elaborating guidelines on experimental designs that take “Sex As a Biological Variable (SABV)” under consideration are available via the Global Preclinical Data Forum. There, it is also stressed that pharmacokinetic and pharmacodynamic differences should also be taken into consideration when investigational doses are selected for both sexes. The serotonergic system is very much sex-differentiated, and sex differences in behavioural outputs as well as stress/fear animal models have been repeatedly reported (Hodes et al., 2023; Kokras and Dalla, 2014). Depression, PTSD and anxiety disorders show varying prevalence between sexes (Labaka et al., 2018; Christiansen and Berke, 2020; Farhane-Medina et al., 2022), thus research in both sexes is imperative, in order to enhance the translational value of the preclinical findings and develop better treatments.

## 3 Neuroimaging methods

### 3.1 Positron Emission Tomography

#### 3.1.1 Dose selection

Positron Emission Tomography (PET) is a powerful tool that leverages radiolabelled compounds to map neurochemical properties of the brain (Heurling et al., 2017). The establishment of the drug occupancy curve with PET brain scanning in humans is the state-of-the-art method to establish which dose to use in subsequent clinical trials (Gunn and Rabiner, 2017). With the right study design and quantification methods, tight correlations between plasma concentration and occupancy can be obtained (Laurell et al., 2022). Occupancy studies demonstrate blood-brain barrier permeability, verify interaction with target and determine the drug dose or plasma concentration resulting in 50% occupancy (ID50 and IC50, respectively). The studies are not inexpensive, but given the risk of failure of subsequent trials if a wrong dose is chosen, it is worthwhile to do, when feasible. This can be applied in psychedelic science e.g., estimating the dose-occupancy relationship at targets of interest, e.g., 5-HT2AR using the radioligand [11C]Cimbi36 (Ettrup et al., 2016). Such occupancy studies will be especially important when comparing novel “non-psychedelic” 2A agonists currently being in development, and classical psychedelics. This strategy is highlighted by what is to date the only plasma concentration-occupancy psychedelic study, estimating psilocybin occupancy of brain 5-HT2AR (Madsen et al., 2019). Psychedelics are pharmacologically promiscuous and further characterization of binding to other receptors in humans in vivo may reveal additional, relevant mechanisms. For example, many psychedelics bind to the inhibitory serotonin 1A receptor (5-HT1AR), which can be measured with [11C]WAY100635 or [11C]CUMI-101 PET (Forster et al., 1995). Currently, only one new occupancy study is ongoing, measuring the occupancy at a range of doses of LSD at 5-HT2AR using [11C]Cimbi36 using simultaneous PET-MR (https://aspredicted.org/gn3un.pdf). Although less tightly associated with plasma psilocin levels, subjective drug intensity has been shown to be related to the occupancy of psilocin at the 5-HT2AR (Madsen et al., 2019), and thus, future drug-development programs may consider this measure in lieu of PET-occupancy studies if these are not available. Such an approach is obviously not applicable for novel psychedelic-based compounds with muted or absent subjective drug effects. Novel psychedelics may exhibit different relations between 5-HT2AR occupancy and subjective drug intensity depending on, e.g., non-2A receptor binding or partial/biased 5-HT2AR agonism. Finally, an evaluation of direct target engagement is advised by regulatory bodies such as the FDA (See FDA brochure “Population Pharmacokinetics Guidance for Industry” Section A. 1 published February 2022), supporting the regulatory relevance of PET occupancy studies for novel compounds.

#### 3.1.2 Neurotransmitter release

PET can also be applied to measure endogenous neurotransmitter release following a stimulus, either a psychedelic or a known releaser such as amphetamine. Using a range of radiotracers, serotonin release ([11C]Cimbi36) (Erritzoe et al., 2020), dopamine release ([11C]raclopride or [11C]PHNO) (Shotbolt et al., 2012) and endorphin release ([11C]carfentanil) (Colasanti et al., 2012; Turton et al., 2020) can be determined. The only PET study to evaluate neurotransmitter release following psychedelic administration showed psilocybin-induced dopamine release using the dopamine D2 radiotracer [11C]raclopride (Vollenweider et al., 1999). This intriguing study should not only be replicated and expanded to other neurotransmitters, but also possibly expanded to evaluate the dose-effect relation. For example, this evaluation of endogenous neurotransmitter release can be used to determine if a patient has impaired serotonin release and then it can be established whether patients with impaired serotonin release, e.g., in depression (Erritzoe et al., 2022), respond better or worse to psychedelic therapy. Psychedelics have shown promise in the treatment of addictive disorders (Bogenschutz et al., 2022; Johnson et al., 2017), disorders in which dopamine and endogenous opioid release are highly relevant (Turton et al., 2020; Mick et al., 2016). Such imaging could also be performed in the period after psychedelic therapy to determine whether psychedelic therapy can restore healthy monoaminergic function in these patients.

#### 3.1.3 Acute metabolic effects

Although early work has been performed, the acute metabolic effects of psychedelics on the brain also requires further characterisation. [18F]FDG PET is the most common clinical PET radiotracer, applied as a marker of glucose metabolism. Two early studies show that psilocybin increases glucose metabolism globally and, after normalising to the global metabolic rate of glucose, relatively lower glucose metabolism was observed in subcortical and occipital brain regions (Gouzoulis- Mayfrank et al., 1997; Vollenweider et al., 1997, Vollenweider et al., 1998). Further work would assist in validating these findings using modern techniques and expand them using multimodal neuroimaging to delineate the relation between e.g., fMRI and FDG PET measures.

#### 3.1.4 Persisting effects on synaptic density

As mentioned above, a prominent theory posits that enhanced neuroplasticity and/or synaptogenesis is an underlying mechanism supporting the persisting effects of psychedelics (Olson, 2018). This hypothesis has so-far not been tested in humans, but only in non-human models of depression-like behaviour (Kwan et al., 2022). The PET radiotracer [11C]UCB-J is one among other PET radiotracers that targets the synaptic vesicle protein 2A (SV2A) located presynaptically (Cai et al., 2019). Thus, if psychedelics produce synaptogenesis, one might expect an increase in the PET SV2A specific binding following administration, as was shown with UCB-J autoradiography in pigs (Raval et al., 2021). There are ongoing trials measuring post-psychedelic changes in synaptic density using UCB-J in healthy (NCT03289949), mild cognitive impairment (Amaev et al., 2023) and depressed cohorts (NCT04630964). In addition, novel radiotracers are in development that bind to postsynaptic density protein 95 (PSD95), which could provide a complementary measure of post-synaptic formation (Serrano et al., 2022). Diffusion-weighted MRI can be used to detect microstructural changes in brain anatomy which may be altered following psychedelics (Assaf and Pasternak, 2008).

### 3.2 Functional imaging

#### 3.2.1 Acute haemodynamic effects

Blood Oxygen Level-Dependent Functional Magnetic Resonance Imaging (BOLD fMRI) leverages measurements of blood oxygenation as a way of estimating brain activity with a millimetre spatial resolution and 0.5-1 Hz temporal resolution. At the time of writing, approximately 50 articles using functional magnetic resonance imaging to investigate the effects of psychedelics have been published, mostly focussed on the acute effects (McCulloch et al., 2022). After the administration of high doses, individuals may not be amenable to standard tasks during peak drug effects and thus task-free imaging is best suited in this case. Naturalistic tasks such as music listening, which is the typical setting for psychedelic sessions, or movie-watching can also be employed and may be of added value as naturalistic tasks have been shown to produce more reliable individual neural fingerprints than rest (Finn et al., 2020). While there is a wealth of data concerning the acute effects of psychedelics on functional brain activity, there have been surprisingly few independent replication or hypothesis-testing studies to complement the small hypothesis-generating studies that have been published thus far. The most prevalent hypotheses include the entropic brain/REBUS (Carhart-Harris, 2018; Carhart-Harris and Friston, 2019), thalamic gating (Avram et al., 2021; Moujaes et al., 2023; Müller et al., 2017) and cortical-claustral-cortical (Barrett et al., 2020) models and each of these require additional multi-site testing. These hypotheses can be further tested using multimodal neuroimaging such as simultaneous fMRI-EEG, as has been explored using psilocybin and DMT (Duerler et al., 2022; Timmermann et al., 2023).

#### 3.2.2 Persisting haemodynamic effects

Despite the many studies of acute effect, there have been very few investigations of the persisting effects of psychedelics on functional brain activity, with only two studies reporting changes measured more than 48 hours after drug administration in depressed patients, one of which investigated a subgroup of the other (Daws et al., 2022; Shukuroglou et al., 2023). Two small sample-studies have been published in healthy populations (Barrett et al., 2020; McCulloch et al., 2021). The lack of characterisation of the persisting effects of psychedelics on functional brain activity was highlighted at the meeting as one of the most pressing knowledge gaps in psychedelic functional brain research. Such studies should be designed based on known pathologies within the disorder of interest (e.g., cue reactivity in addiction patients) and also based on indicated lasting psychological effects of psychedelics, e.g., increases in mindful-attention (Søndergaard et al., 2022). Due to the high degrees of interpretive flexibility in fMRI research, it is critically important that researchers pre-register their hypotheses and analyses beforehand, making use of platforms such as ‘as predicted’ (aspredicted.org) and ‘open science framework’ (https://osf.io/). It was highlighted at the meeting that in order to more deeply understand the link between acute and persisting psychedelic effects, it is highly desirable to perform fMRI before, during and after psychedelic sessions in the same patients, despite the technical difficulties that this may pose.

#### 3.2.3 Electroencephalography

Acute and persisting neural effects of psychedelics can also be investigated using electroencephalography (EEG). Task-free EEG has been evaluated during acute psychedelics effects, showing consistent decreases in alpha power (Timmermann et al., 2019, 2023; Carhart-Harris et al., 2016; Kometer et al., 2015; Murray et al., 2022; Muthukumaraswamy et al., 2013; Pallavicini et al., 2021) and increases in Lempel-Ziv complexity (Timmermann et al., 2019, 2023). However, whether psychedelic-induced alterations in alpha-power or signal complexity are related to subsequent symptom reductions is yet to be evaluated. Additionally, future EEG work may seek to use paradigms such as visual long-term potentiation (Teyler et al., 2005) and mismatch negativity (Garrido et al., 2009) to evaluate psychedelic effects on functional plasticity.

## 4 Conclusions

Psychedelics are showing promise as forthcoming medicines for the treatment of several psychiatric disorders. Although evaluations of safety and efficacy required for medical approval and implementation in health systems will be driven by large phase 3 clinical studies, developing a deeper understanding of the molecular and functional mechanisms of action will be imperative for both optimising treatments with current medicines, and in the development of next-generation psychedelic-based compounds with improved safety and efficacy. In this manuscript we have reviewed key knowledge gaps in the mechanism of action of psychedelics. These include effects on animal behaviour, potential pro-plasticity effects, sex differences, dose optimisation, and the acute and persisting effects on functional brain activity and neuropharmacology as can be revealed with clinical PET, fMRI and EEG.

## Declaration of competing interest

DEM salary is supported by an unrestricted grant from COMPASS Pathways Ltd. who had no part in the conceptualisation or writing of this manuscript. DE has scientific advisory roles for, Aya Biosciences Inc., Clerkenwell Health, SmallPharma Ltd (and is CI on their phase I and IIa depression trials) and within last 5 years has received advisor fees from Mydecine, Field Trip Health and Entheon. EC is a cofounder and board member of Kasvu Therapeutics and has received lecture fees from Janssen-Cilag. KHP is currently and employee of Boehringer Ingelheim GmbH & CO KG. VGF discloses that she has received honorarium in form of lecture fees from SAGE Therapeutics, Lundbeck Pharma A/S, Janssen Cilag A/S and Gedeon-Richter A/S. GMK has within the last three years served as a speaker for AbbVie, Angelini, H. Lundbeck and Sage Biogen and an advisor for AbbVie, Gilgamesh, Onsero, Pangea Botanica, PureTec, and Sanos. PMF, JL, JPL and CD declare no conflicts.
